# Thoracic surgery may alter body static balance via diaphragm dysfunction

**DOI:** 10.1371/journal.pone.0273641

**Published:** 2022-08-31

**Authors:** Janusz Kocjan, Bożena Gzik-Zroska, Katarzyna Nowakowska-Lipiec, Michał Burkacki, Sławomir Suchoń, Robert Michnik, Damian Czyżewski, Mariusz Adamek

**Affiliations:** 1 Chair and Department of Thoracic Surgery, Faculty of Medical Sciences, Medical University of Silesia, Katowice, Poland; 2 Department of Biomaterials and Medical Devices Engineering, Faculty of Biomedical Engineering, Silesian University of Technology, Zabrze, Poland; 3 Department of Biomechatronics, Faculty of Biomedical Engineering, Silesian University of Technology, Zabrze, Poland; Stanford University School of Medicine, UNITED STATES

## Abstract

Many diseases and conditions can alter an ability to maintain body balance. The aim of the present study was to investigate whether thoracic surgery may elicit diaphragm dysfunction thereby impairing postural stability. 40 patients qualified to video-assisted thoracoscopy (VATS) lobectomy or lobectomy via thoracotomy due to pulmonary carcinoma were examined two times: a day before lung resection and 3–5 days after surgical procedure. Diaphragm assessment was performed using ultrasonography, while postural sways were evaluated by Zebris FDM-S stabilometric platform. Thoracic surgery was associated with decrease of diaphragm thickness and movement, as well as, with deterioration of static body balance maintenance. Upper lobe resection was linked with greater diaphragm excursion restriction and worse body sway parameters than middle and lower lobe resection. VATS lobectomy was associated with better postoperative diaphragm function and better postural sway parameters than lobectomy via thoracotomy. Patients after lobectomy via thoracotomy had significantly more load on lower limb on the operated side than patients after VATS lobectomy. Impairment of diaphragm function is closely associated with equilibrium impairment after pulmonary resection. VATS lobectomy was less invasive than lobectomy via thoracotomy in terms of primary respiratory muscle function and body balance maintenance parameters.

## Introduction

The diaphragm is a principal muscle involved in the respiratory process and whole body homeostasis. This ability to maintain the internal environment steadiness is a result of coordinated interactions between breathing and other systems (postural, cardiac, lymphatic, gastrointestinal) in human body where the diaphragm plays also an important role [1, 2]. When this mechanism fails due to breathing pattern disorders or diaphragm dysfunction, the human body begins to function suboptimally what may lead to a whole set of symptoms that can be observed in a different areas of the body [3–8]. However, breathing rhythms can be brought under conscious control and thus can improve coupling between body systems providing an avenue for physiological self-regulation and restoring physiological efficiency [9].

Postural control has been defined as the act of maintaining, achieving or restoring a state of balance during any posture or activity [10]. Many conditions and diseases can disturb ability to postural stability [11–14]. To this time, only one previous study investigated postural stability in patients after lung resection. Mraz et al., observed a shift of the position of the body’s center of gravity and significant alteration of postural stability which caused an asymmetrical (left or right-sided) posture during balance maintenance in the upright standing position. The authors concluded that these effects are results of a violation of the chest wall musculature corset due to the surgical procedure or as a result of postoperative pain syndromes. They suggested that patients subconsciously try to protect themselves against this pain. This situation leads to overloading of the musculoskeletal system what in consequences provoke disorders of the body posture [15].

However, still a little is known about other factors contributing to balance disorders after thoracic surgeries, especially associated with postoperative diaphragm dysfunction. In the present paper, we focused on the clinical aspects of lung resection trying to find the answer to the following research questions:

1. Does diaphragm dysfunction after surgery can disturb the postural sway?
2. Do the values of the diaphragm function parameters and postural sway parameters after lung resection depend on the kind of surgery approach?
3. Is there a relationship of lung resection area with the diaphragm function and body sway parameters?
4. Are there differences in diaphragm function and balance parameters between operated and non-operated side?

## Material and methods

### Participants

64 pulmonary carcinoma patients qualified to lobectomy at our department were enrolled to the study. We excluded from the study patients who had: concomitant diseases that can disturb the balance maintenance (neurological, laryngological, orthopedical), previous lobe resection due to lung cancer, previous thoracic or abdominal surgery. We excluded also from postoperative measurements patients who had: poor general well-being after surgery and was not fit to perform the second measurement (strong postoperative pain, significant weakness, dizziness), chest tube in the pooperative measurement day, postsurgery complications. We also had a random loss of a patient when he was discharged from the hospital on the day we were unable to take postoperative measurements (ie saturday or sunday). Having implemented the exclusion criteria, a total of 40 patients who fitted the criteria were included into the study and were examined two times: a day before lung resection and 3–5 days after surgical procedure. Mean subjects age was 63.74+11.87 (range 42–78). Gender composition was 62.5% (N = 25) males versus 37.5% (N = 15) females. 57.5% (N = 23) of patients underwent standard thoracotomy, while 42.5% (N = 17) of them were subjected to videothoracoscopy. The highest percentage of individuals had lower lobe resection (27.5% left and 25% right). 20% had right upper resection, 17.5% left upper and 10% right middle. In most cases (55%) the right side of the chest was operated.

### Surgical techniques

Any patient with enlarged mediastinal lymph nodes on CT scan had endobronchial ultrasound (EBUS) performed before surgery. In case of positive lymph nodes, the patients received neoadjuvant treatment. All the excised lymph nodes were examined histopathologically.

All patients were operated by well-trained surgeons with long experience who worked at our thoracic surgery ward. Both procedures were performed under general anesthesia with a single lung ventilation. A complete radical lymphadenectomy was performed in all patients. The subjects were placed on a lateral decubital position. Pulmonary resection via open conventional thoracotomy were performed through the muscle-sparing posterolateral incision using the 5th or 6th intercostal spaces. In the VATS group, all patients were operated using uniportal approach with no rib spreading. The 3–5 cm single incision was placed in the 5th intercostal space. The scope camera was introduced in the upper part of the incision. After completion of the resection, the chest tube was inserted in the posterior part of the incision and was sutured to the anterior and posterior margins of the uniportal skin incision.

A complete radical lymphadenectomy was performed in all patients. Lymph nodes (LNs) 7, 10 and 11 were always resected. Additionally, we removed LNs 4R (upper right lobectomy), LNs 5 (upper left lobectomy), LNs 8 and 9 (lower lobectomy). Any of the mediastinal lymph nodes was further than 2 cm from the phrenic nerve. Furthermore, during lobectomy the surgeons did not use electrocoagulation in the area of the phrenic nerve, which would cause damage or even temporary paralysis of the phrenic nerve.

### Diaphragm muscle assessment

The diaphragm muscle contractile activity was measured using ultrasonography (ALOKA ultrasound machine). Patients were examined in supine position by trained specialist who was blinded to balance measurements results. Two approaches were used to visualize the diaphragm. Brightness mode (B-Mode) to determine diaphragm thickness at the zone of apposition. In this case the high frequency (9–12 MHz) linear probe was placed at the mid-axillary line between 8th and 10th rib. Motion mode (M-Mode) was used to evaluate the diaphragm excursion during quiet breathing, deep breathing and voluntary sniffing in real time. To obtain a diaphragm picture, the liver or spleen acoustic window was used for right and left dome, respectively. The curvilinear low frequency transducer (1–4 MHz) was placed in the subcostal area between the midclavicular and anterior axillary lines, and was directed cranially, medially and tilted dorsally [16]. All ultrasound measurements were performed three times and the average value was included to statistical analysis.

Following diaphragm parameters were evaluated:

**ThIns (Th**ickness **Ins**piratory**)**—diaphragm thickness measured at the maximal inspiration estimated as the vertical distance between the pleural and peritoneal layers
**ThExp (Th**ickness **Exp**iratory**)**—diaphragm thickness measured at the end of maximal expiration estimated as the vertical distance between the pleural and peritoneal layers
**DTF (D**iaphragm **T**hickness **F**raction**)**—percentage ratio reflecting the magnitude of diaphragmatic effort computed from formula: (ThIns–ThExp)/ThExp*100%.
**Diaphragm Excursion**—diaphragm movement represented as a tracing echogenic line moves during breathing cycles. Excursion was estimated as the vertical distance between the baseline to the peak of the tracing line during quiet breathing (QB), deep breathing (DB) and sniff maneuvers (fast nose breath).
**DEA (D**iaphragm **E**xcursion **A**mplitude**)—**differences in diaphragm excursion between two measurements calculated from formula: DEA = preoperative value—postoperative value.
%**DEA (% D**iaphragm **E**xcursion **A**mplitude**)—**percentage differences in diaphragm excursion between two measurements calculated from formula: %DEA = (preoperative value—postoperative value)/postoperative value*100%.
**Modul DEA** (**Modul** of **D**iaphragm **E**xcursion **A**mplitude)—mean difference of between preoperative and postoperative values of left and right diaphragm dome, calculated from formula: Modul DEA = [(postoperative value-preoperative values of left dome)+(postoperative value-preoperative values of right dome)/2.
**S-T-S-V (S**ide-**T**o-**S**ide-**V**ariation**)—**percentage differences between left and right hemidiaphragm excursion. Difference more than 50% is interpreted as one-side diaphragm paralysis

### Balance maintenance assessment

The static balance maintenance assessment was carried out on multifunction force distribution measuring plate system Zebris FDM-S (Zebris Medical GmbH, Germany). Following body sway parameters were analysed: ellipse area, path length, left and right side load, hind and forefoot load. All these parameters were assessed during the Romberg test (standing position with eyes closed and open for 30 seconds). The participants were positioned in bipedal standing with a slight stride, with the knees extended, feet situated parallel and heels aligned in a line. During stances with the eyes open, the eyesight was focused on the wall at eye level 3 meter from the volunteer. The pressure distribution under the feet was monitored on the computer. Each measurement was repeated three times and average values was take into the analysis. At the base of obtained data we also calculated: L-P Modul (difference between left and right side load), F-H Modul (difference between forefoot and hindfoot load) and Balance Amplitude (difference between postoperative and preoperative values).

### Statistical analysis

Data included in the analysis were collected from January 2017 to July 2018. The Statistica StatSoft Software (version 12.0) was used to prepare the statistical analysis. To test the assumption of normal data distribution the Kolmogorov-Smirnov test was used. Descriptive statistics are presented as the mean [M] ± standard deviation [SD], frequencies [%] and 95% Confidence Interval [95%CI]. To compare the baseline characteristics (preoperative) and clinical outcomes (postoperative) the Wilcoxon signed rank test was applied. Pearson correlations were performed to evaluate associations between continuous variables. Statistical significance was asserted at p level less than 0.05.

### Ethics approval

The study was approved by the ethical committee of Silesian Medical University in Katowice, Poland (Decision number: KNW 88444663) and was conducted in accordance with the amended Declaration of Helsinki for human research. All participants were informed about the procedures they would undergo and gave their written consent to voluntary participation in the study.

## Results

**Fig 1:**
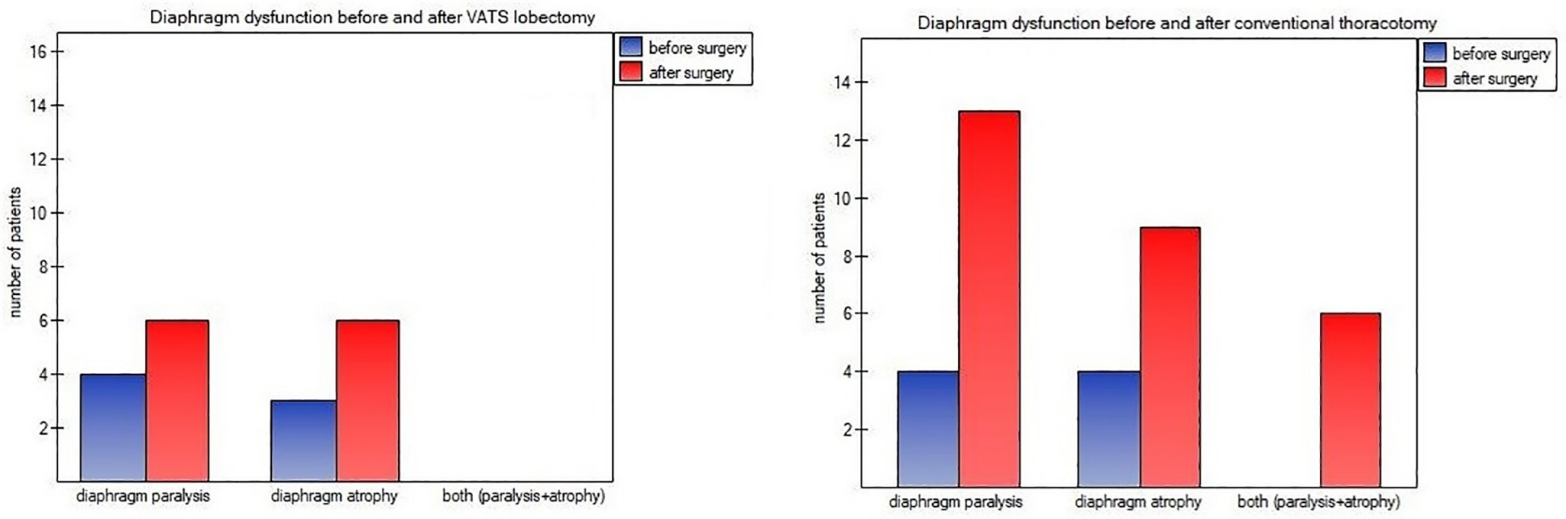
Percentage of patients with diaphragm dysfunction before and after surgery.

We demonstrated a postoperative decrease of inspiratory thickness and diaphragm thickness fraction comparison to preoperative values, regardless to surgical approach. Mean values were as follows: {VATS} ThIns 0.27±0.04 (95%CI:0.25–0.29) vs 0.31±0.05 (95%CI:0.28–0.33), p<0.001; DTF 24.25±12.87 (95%CI: 18.99–29.34) vs 35.98±20.94 (95%CI: 28.19–45.31), p = 0.007; {Thoracotomy} ThIns 0.27±0.03 (95%CI:0.25–0.29) vs 0.30±0.04(95%CI:0.28–0.32), p<0.001; DTF 22.88±14.51 (95%CI: 18.56–25.63) vs 36.56±22.71 (95%CI: 29.12–43.94), p<0.001. No differences were found in expiratory thickness between two measurements: {VATS} 0.21±0.06 (95%CI: 0.19–0.22) vs 0.22±0.04 (95%CI: 0.21–0.23), p = 0.098; {Thoracotomy} 0.21±0.05 (95%CI: 0.19–0.24) vs 0.22±0.05 (95%CI: 0.20–0.24), p = 0.105. All patients had worse postoperative values of inspiratory thickness and diaphragm thickness fraction compared to preoperative data. Worsening of expiratory thickness after VATS method was noted among 17,6% (n = 3) of participants, while 82,4% (n = 14) of them had identical values as in preoperative measurements—compared to 17,4% (n = 4) and 82,6% (n = 19) after conventional thoracotomy, respectively. The percentage of patients with preoperative and postoperative diaphragm atrophy (defined as ThExp values less than 2mm), diaphragm paralysis (defined as DTF value less than 20%) and both types disorders are presented in Fig 1.

Results of sonographic study showed a reduce of diaphragm excursion during spontaneous and deep breathing, as well as sniff test after thoracic surgery—relative to output data, in both surgical procedures. All analysed patients had worse postoperative diaphragm kinetics during all analysed breathing manoeuvres. Significantly greater impairment of diaphragm motion measured as DEA (Diaphragm Excursion Amplitude) and %DEA (Percentage of Diaphragm Excursion Amplitude) was reported after lobectomy via thoracotomy than VATS. Detailed findings are presented in Table 1.

**Table 1:** Descriptive statistics of diaphragm excursion.

*Variables*		QB right	QB left	DB right	DB left	Sniff right	Sniff left
**VATS**	Preoperative	1.97±0.41	1.99±0.46	4.72±1.08	4.90±1.18	2.30±0.56	2.53±0.56
		(1.84–2.02)	(1.93–2.03)	(4.42–5.08)	(4.49–5.38)	(2.14–2.49)	(2.31–2.69)
	Postoperative	1.48±0.36	1.52±0.41	3.83±0.99	3.92±0.91	2.15±0.42	2.17±0.38
		(1.36–1.54)	(1.46–1.58)	(3.55–4.11)	(3.63–4.36)	(1.96–2.28)	(2.03–2.36)
	p value	**<0.001**	**0.008**	**0.032**	**0.008**	**<0.001**	**0.002**
	DEA	0.18±0.46**	0.14±0.52*	0.69±1.10**	0.59±1.09**	0.19±0.45*	0.13±0.37**
	% DEA	9.52**	8.47**	14.89**	12.66**	10.46**	9.98**
**THORACOTOMY**	Preoperative	1.94±0.39	1.96±0.43	4.65±1.14	4.99±1.12	2.34±0.57	2.50±0.55
		(1.87–2.06)	(1.91–2.02)	(4.40–4.95)	(4.62–5.42)	(2.16–2.51)	(2.36–2.66)
	Postoperative	1.40±0.32	1.44±0.38	3.64±0.90	3.71±0.88	1.96±0.34	2.12±0.37
		(1.24–1.55)	(1.35–1.47)	(3.49–3.77)	(3.47–4.22)	(1.88–2.07)	(1.97–2.28)
	p value	**<0.001**	**<0.001**	**<0.001**	**<0.001**	**<0.001**	**<0.001**
	DEA	0.25±0.53**	0.20±0.51*	0.91±1.21**	0.77±1.18**	0.25±0.39*	0.23±0.36**
	% DEA	16.11**	14.36**	19.91**	17.74**	12.33**	11.89**

**Note**: DEA and %DEA values statistically significant between VATS and Thoracotomy at p level:

*<0.05;

**<0.01

**Abbreviations**: QB (quiet breathing), DB (deep breathing), DEA (Diaphragm Excursion Amplitude), %DEA (% Diaphragm Excursion Amplitude)

**Fig 2:**
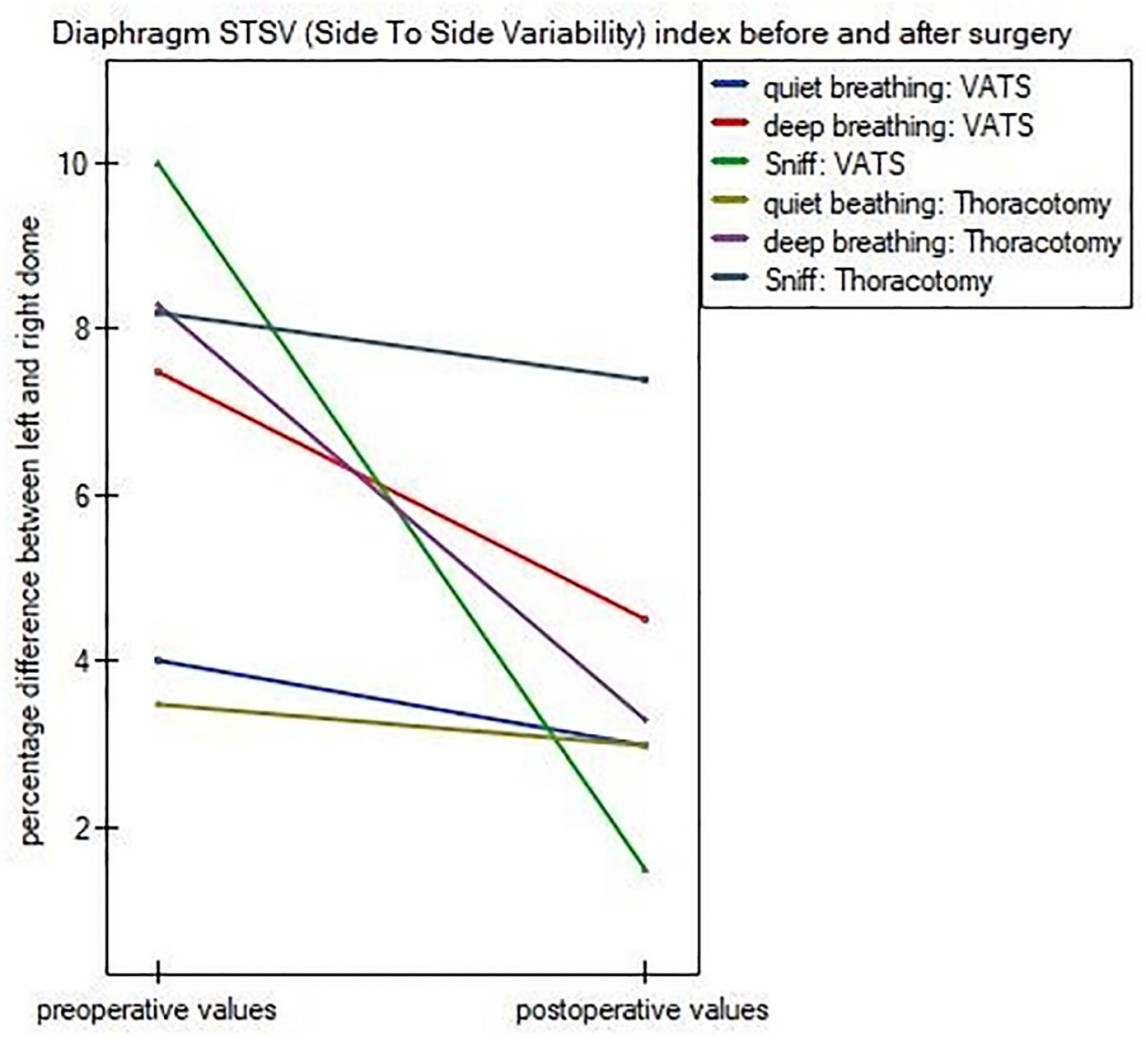
Diaphragm STSV (Side To Side Variability) index before and after surgery.

Only one from forty patients had postoperative difference of movement between cupolae (side-to-side-variability index) more than 50% during all breathing maneuvers, what indicated for one side diaphragm paralysis. We found no differences between patients undergone vats and conventional lobectomy in range of preoperative (QB: p = 0.884; DB: p = 0.636; Sniff: p = 0.246) percentage mobility of left and right domes. In case of postsurgery values only differences in sniff maneuver were significant (QB: p = 0.907; DB: p = 0.424; Sniff: p = 0.001). In both groups the postoperative values of STSV index were lower than preoperative (QB: p = 0.605, p = 0.768; DB: p = 0.261, p = 0.011; Sniff: p<0.001, p = 0.864; VATS vs Thoracotomy, respectively). Detailed data are given in Fig 2.

**Fig 3:**
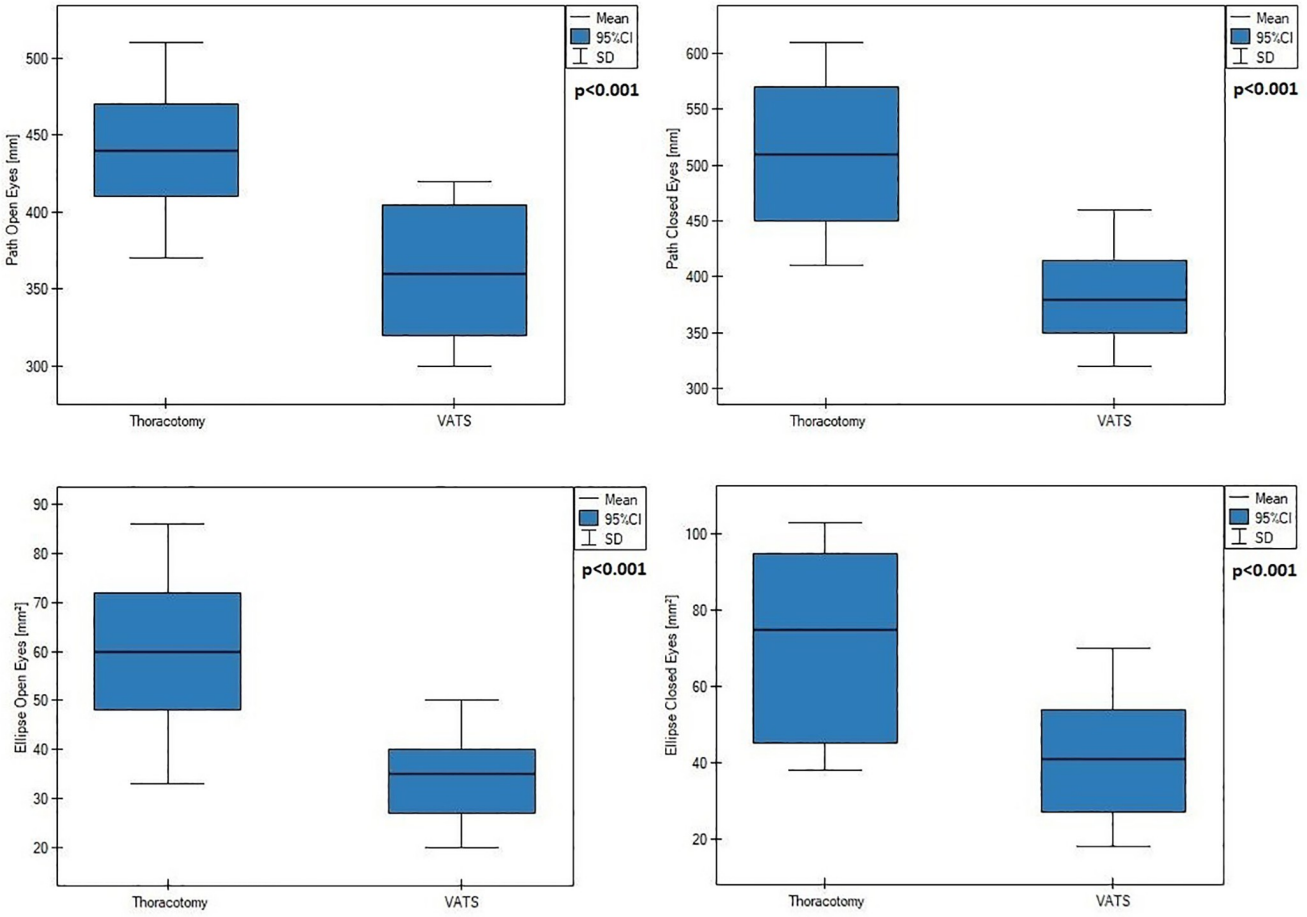
Postoperative values of path length and ellipse field after VATS and thoracotomy.

**Fig 4:**
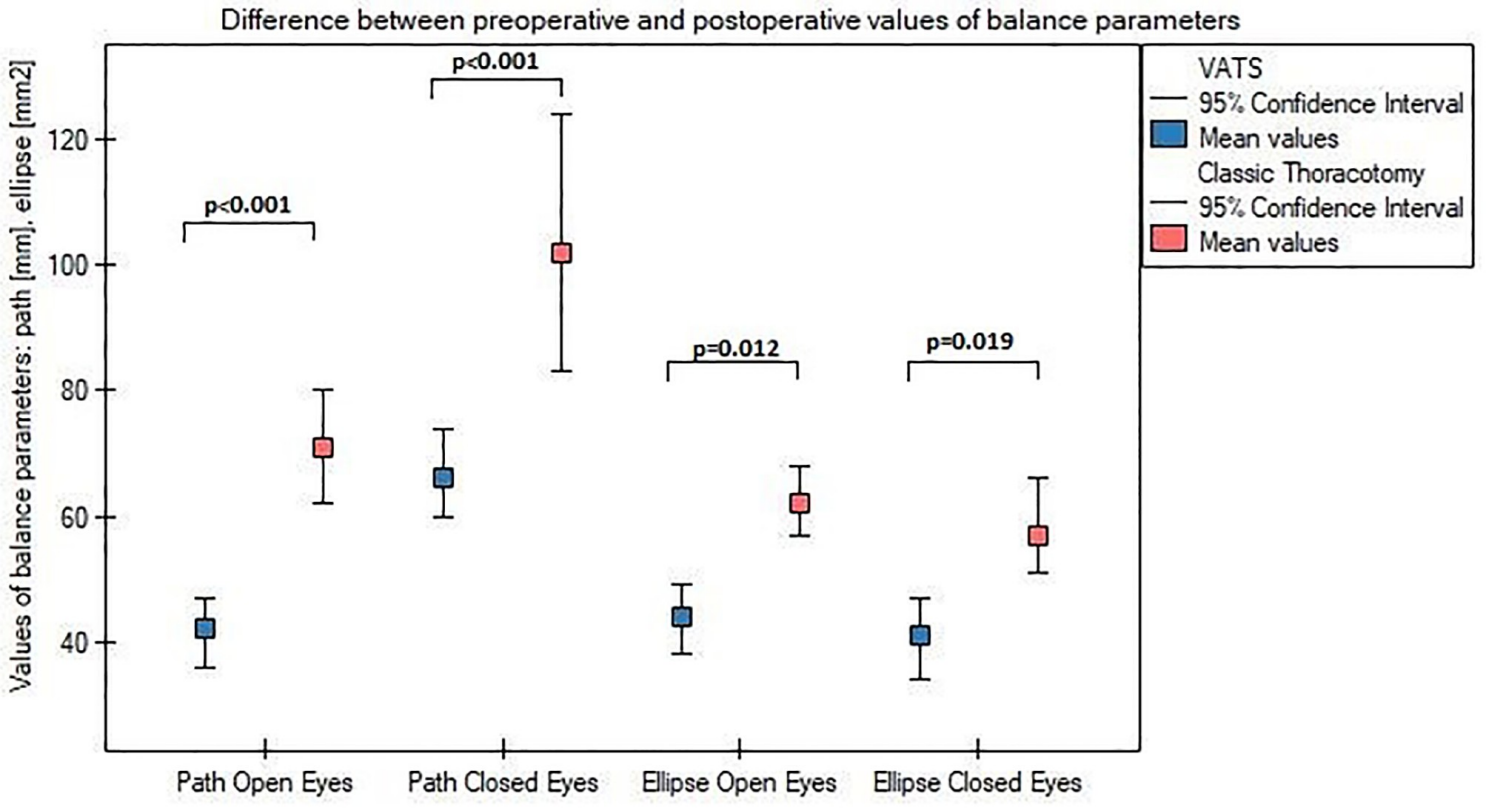
Mean deterioration of path length and ellipse field after VATS and thoracotomy.

Analysing baseline data, patients qualified to VATS lobectomy and conventional lobectomy had similar values of balance parameters (p value > 0.05). Both surgical procedures were associated with significant (p<0.01) deterioration of two main balance parameters: path length and ellipse field, measured with open and closed eyes. Patients underwent classic thoracotomy had significantly worse postoperative values of path length and ellipse field (Fig 3), as well as characterized greater deterioration of this two parameters, assessed as difference between postoperative and preoperative values (Fig 4)—compared to patients underwent VATS method.

Correlational analysis revealed a significant relationship between diaphragm excursion amplitude and path length (Table 2). We found no association of diaphragm excursion amplitude with load on the left and right side of the body, as well as the load on the forefoot and hindfoot.

**Table 2:** Correlation coefficients between diaphragm excursion amplitude and balance Amplitude.

Variables		modul: Path OE	modul: Path CE	Modul: Ellipse OE	Modul: Ellipse CE
Modul DEA: Quiet Breathing	VATS	**-0,493** *	**-0,386** *	-0,224	-0,316
	Thoracotomy	**-0,516** *	**-0,427** *	-0,237	-0,324
Modul DEA: Deep Breathing	VATS	-0,586**	**-0,441** *	-0,242	-0,294
	Thoracotomy	**-0,622** **	**-0,426** *	-0,271	-0,277
Modul DEA: Sniff	VATS	**-0,539** *	**-0,402** *	-0,258	-0,337
	Thoracotomy	**-0,545** *	**-0,424** *	-0,266	-0,306

Note:

*p<0.05;

**p<0.01.

Abbreviations: OE: Open Eyes; CE: Closed Eyes; Modul DEA: Modul of diaphragm excursion amplitude

**Fig 5:**
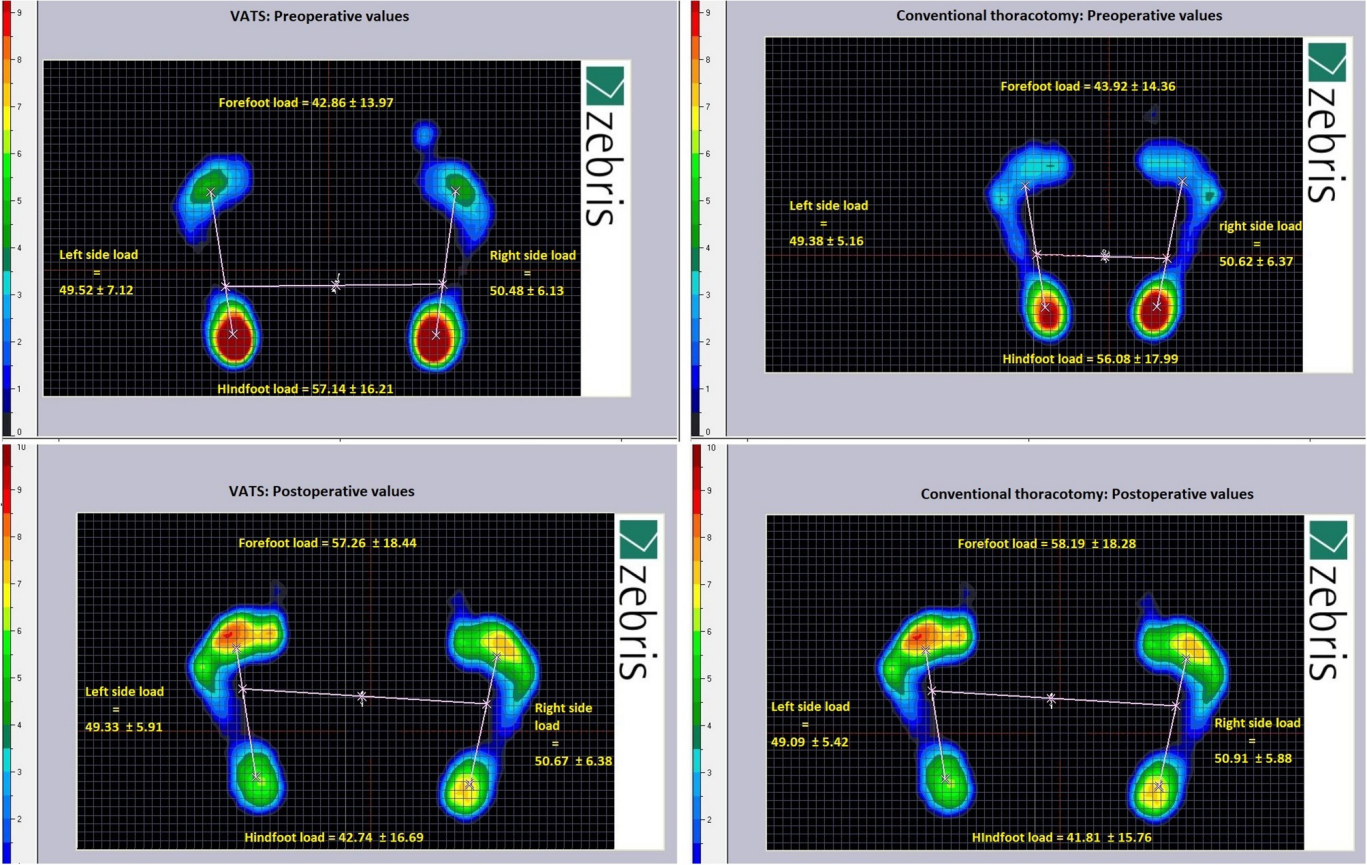
Preoperative and postoperative load of hindfoot and forefoot.

Lung resection also caused disturbances in the body statics in terms of load transmission through the forefoot and hindfoot. Patients qualified to surgery, were characterized by a greater load on the hindfoot than forefoot. Inverse results were noted postoperatively—a greater load on the forefoot relative to the hindfoot was demonstrated. There was no differences on the left and right foot load in both groups (Fig 5).

**Fig 6:**
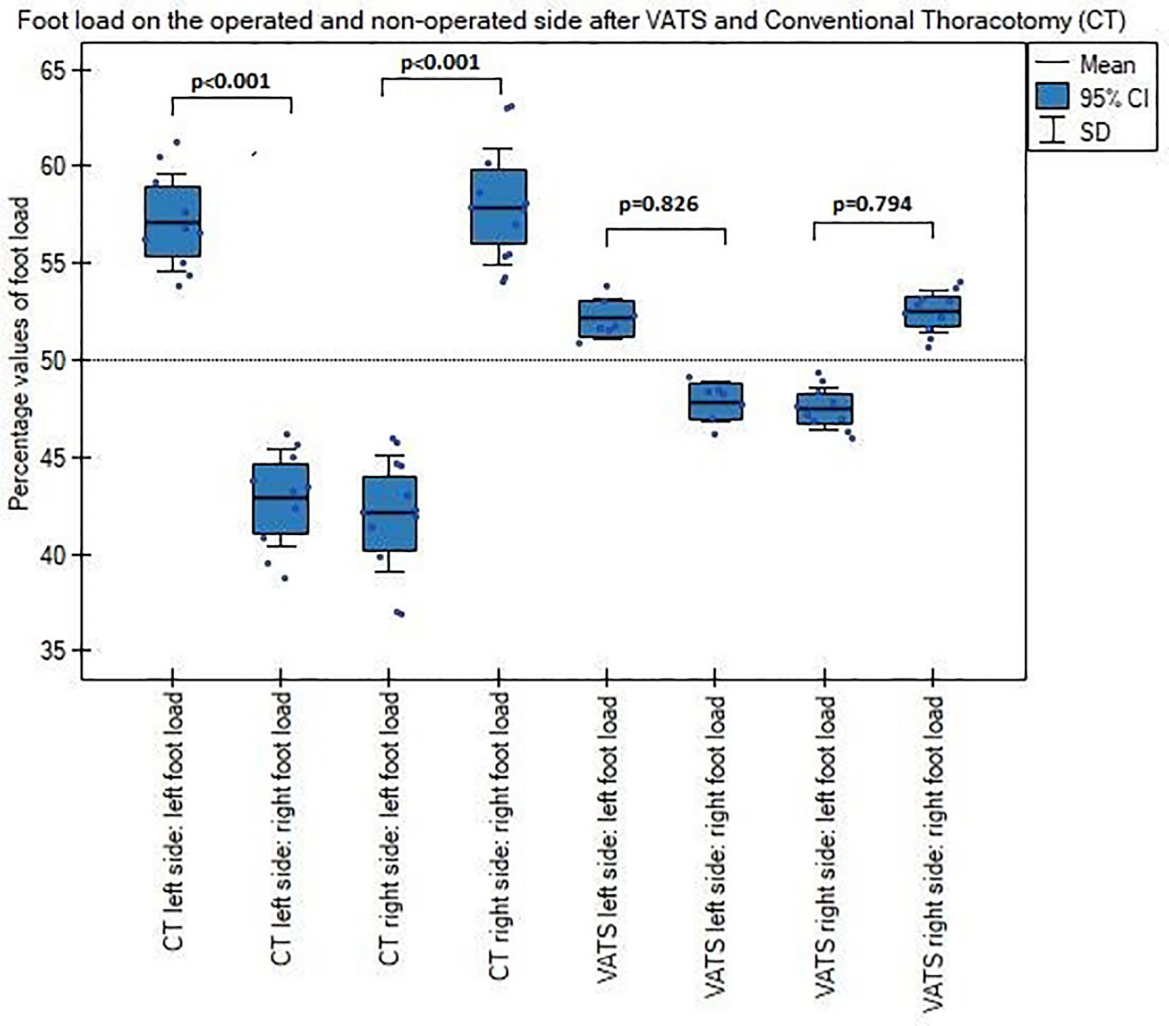
Left and right foot load on the operated and non-operated side with open eyes after VATS and thoracotomy.

**Fig 7:**
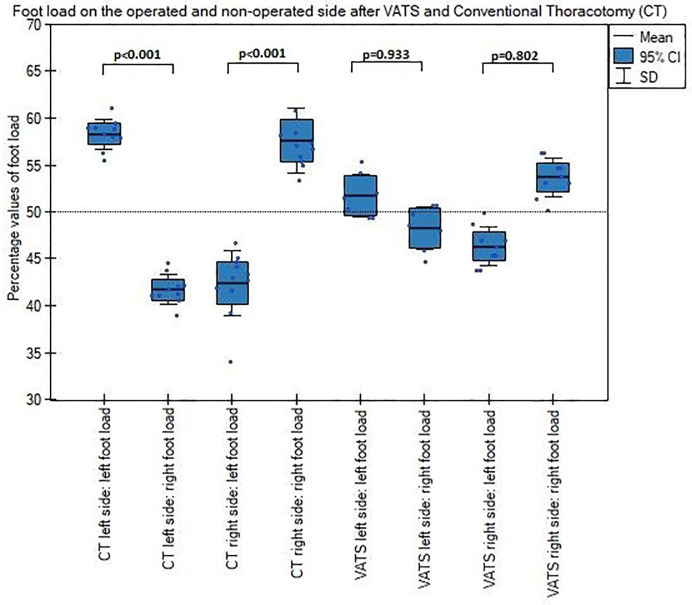
Left and right foot load on the operated and non-operated side with closed eyes after VATS and thoracotomy.

Patients after conventional thoracotomy put more pressure on the lower limb on the operated side than non-operated side, both with open and closed eyes and had significantly greater left and right foot load difference after surgery than patients underwent VATS lobectomy (Figs 6 and 7).

Values of diaphragm excursion and main postural sway parameters divided into area of lung resection are given in Tables 3 and 4, respectively.

**Table 3:** Diaphragm excursion during various breathing maneuvers related to area of resection.

variables		QB left [cm]	DB left [cm]	Sniff left [cm]	QB right [cm]	DB right [cm]	Sniff right [cm]
Upper right lobe resection (n = 8)	before	1.98±0.43 (1.83–2.01)	4.95±1.19 (4.61–5.24)	2.49±0.54 (2.26–2.73)	1.96±0.42 (1.86–2.02)	4.92±1.16 (4.66–5.14)	2.46±0.52 (2.27–2.69)
	after	1.40±0.41 (1.29–1.53)	3.72±1.15 (3.46–3.98)	2.09±0.52 (1.96–2.20)	1.39±0.39 (1.27–1.54)	3.68±1.11 (3.40–3.95)	2.02±0.46 (1.89–2.12)
	**p value**	**<0.001**	**<0.001**	**<0.001**	**<0.001**	**<0.001**	**<0.001**
	DEA	0.23±0.26	0.71±0.40	0.20±0.11	0.22±0.27	0.67±0.33	0.22±0.13
Middle right lobe resection (n = 4)	before	1.96±0.42 (1.82–2.06)	4.90±1.12 (4.54–5.18)	2.52±0.56 (2.27–2.77)	1.93±0.43 (1.78–2.06)	4.86±1.14 (4.67–5.02)	2.50±0.53 (2.24–2.71)
	after	1.50±0.36 (1.37–1.64)	3.86±1.04 (3.52–4.14)	2.15±0.50 (1.97–2.32)	1.47±0.38 (1.32–1.59)	3.82±1.04 (3.57–4.13)	2.11±0.46 (1.95–2.30)
	**p value**	**<0.001**	**0.008**	**0.001**	**0.003**	**0.012**	**<0.001**
	DEA	0.20±0.34	0.65±0.38	0.18±0.08	0.19±0.33	0.66±0.35	0.20±0.12
Lower right Lobe Resection (n = 10)	before	1.94±0.44 (1.80–2.05)	5.01±1.15 (4.72–5.32)	2.53±0.53 (2.26–2.68)	1.91±0.41 (1.74–2.06)	4.97±1.10 (4.74–5.23)	2.49±0.50 (2.29–2.70)
	after	1.49±0.40 (1.35–1.66)	3.91±1.03 (3.70–4.16)	2.16±0.48 (2.00–2.31)	1.48±0.37 (1.33–1.68)	3.79±1.03 (3.51–4.02)	2.12±0.45 (1.98–2.26)
	**p value**	**<0.001**	**0.006**	**0.001**	**0.004**	**0.007**	**<0.001**
	DEA	0.18±0.29	0.68±0.33	0.19±0.10	0.17±0.30	0.70±0.38	0.17±0.09
Upper center lobe Resection (n = 7)	before	1.97±0.43 (1.79–2.05)	4.92±1.14 (4.56–5.23)	2.52±0.52 (2.24–2.71)	1.95±0.39 (1.80–2.11)	4.90±1.12 (4.52–5.26)	2.49±0.51 (2.20–2.71)
	after	1.43±0.39 (1.30–1.51)	3.76±1.08 (3.54–3.97)	2.08±0.47 (1.94–2.26)	1.41±0.36 (1.32–1.53)	3.72±1.06 (3.50–3.97)	2.01±0.44 (1.82–2.21)
	**p value**	**<0.001**	**<0.001**	**<0.001**	**<0.001**	**<0.001**	**<0.001**
	DEA	0.19±0.31	0.74±0.36	0.21±0.11	0.20±0.32	0.72±0.39	0.22±0.12
Lower center lobe resection (n = 11)	before	1.97±0.45 (1.82–2.09)	4.93±1.09 (4.55–5.24)	2.54±0.58 (2.27–2.70)	1.94±0.44 (1.76–2.13)	4.89±1.11 (4.57–5.21)	2.51±0.53 (2.26–2.73)
	after	1.50±0.41 (1.35–1.63)	3.83±1.09 (3.62–4.08)	2.15±0.56 (2.02–2.30)	1.48±0.39 (1.32–1.65)	3.80±1.01 (3.58–4.06)	2.13±0.45 (2.01–2.29)
	**p value**	**<0.001**	**0.011**	**0.001**	**0.003**	**0.010**	**<0.001**
	DEA	0.23±0.28	0.67±0.31	0.20±0.12	0.22±0.34	0.65±0.35	0.18±0.08

**Table 4:** Values of path length and ellipse field related to area of resection.

Variables		Path length OE [mm]	Ellipse Field OE [mm2]	Path Length CE [mm]	Ellipse Field CE [mm2]
Upper right lobe resection (n = 8)	Before	456.53±169.56 (412.64–506.93)	75.56±44.37 (66.24–86.27)	603.63±400.91 (551.14–662.44)	101.95±89.57 85.16–122.69)
	After	541.96±159.06 (488.75–594.67)	134.33±75.12 (116.77–150.94)	698.78±318.77 (588.71–764.46)	182.48±103.77 (153.48–211.33)
	**p value**	**0.041**	**0.037**	**0.048**	**0.041**
	Amplitude	62.37±81.16	67.18±35.57	77.89±56.11	74.83±41.15
Middle right lobe resection (n = 4)	before	365.46±42.26 (308.16–393.74)	101.70±62.17 (90.14–113.45)	538.93±102.28 (472.56–607.16)	161.50±81.93 (147.83–176.80)
	after	424.86±38.07 (363.44–478.83)	167.03±71.04 (153.49–201.92)	598.53±300.91 (526.78–665.56)	199.66±99.95 (165.78–234.57)
	**p value**	0.126	0.405	0.142	0.668
	Amplitude	53.42±51.67	39.35±24.57	56.71±43.68	16.56±9.71
Lower right Lobe Resection (n = 10)	before	296.05±42.24 (253.66–345.21)	46.08±20.38 (40.11–53.63)	402.76±74.68 (348.97–470.22)	59.06±61.89 (52.81–69.68)
	after	314.84±76.30 (267.86–361.28)	84.42±21.16 (41.18–68.29)	429.75±117.38 (342.36–502.77)	59.96±42.53 (51.34–68.76)
	**p value**	0.483	0.207	0.416	0.980
	Amplitude	41.71±32.45	20.14±7.88	24.37±14.36	2.80±1.14
Upper Left lobe Resection (n = 7)	before	499.53±141.87 (467.83–538.95)	109.45±88.70 (98.09–124.51)	615.68±79.29 (564.27–677.20)	101.82±65.88 (93.47–109.88)
	after	598.10±91.51 (507.89–669.03)	267.56±94.49 (232.44–297.97)	761.94±201.08 (673.22–840.50)	188.04±110.79 (165.38–210.96)
	**p value**	**0.037**	**0.008**	**0.013**	**0.036**
	Amplitude	67.84±78.95	82.15±63.78	96.84±78.77	77.27±40.56
Lower left lobe resection (n = 11)	before	325.77±36.68 (294.64–361.37)	60.45±24.64 (51.12–70.45)	542.69±181.54 (500.29–588.14)	111.08±51.88 (102.46–126.71)
	after	393.90±93.74 (331.17–449.09)	78.52±80.90 (69.87–88.92)	549.67±211.49 (482.38–617.54)	142.19±65.12 (124.61–157.82)
	**p value**	0.329	0.241	0.930	0.658
	Amplitude	47.93±40.82	13.47±8.01	14.54±6.22	17.12±13.47

## Discussion

Video-assisted thoracic surgery (VATS) and thoracotomy are widely used lobectomy procedures for the treatment of early stages of a non-small cell lung cancer. It is generally agreed that VATS approach has more advantages over the conventional thoracotomy in terms of shortened length of hospital stay, drainage time, bleeding time, decreased of postoperative pain and morbidity, less incisional pain, as well as, safety, feasibility, and a better quality of life [17–21]. Minimally invasive surgery is superior as entailing a better preservation of chest muscles and respiratory function. A smaller decrease in value of Maximal Inspiratory Pressure (MIP) and Maximal Expiratory Pressure (MEP), as well as Forced Expiratory Volume (FEV) and Vital Capacity (VC) was reported among patients undergoing thoracoscopy or video assisted thoracic surgery than thoracotomy [22, 23]. A few studies evaluated diaphragm function after thoracic surgeries. In an animal model, the shortening of diaphragm fraction decreases both after VATS and open thoracotomy, but faster recovery was observed after VATS [24]. Fratacci noted a marked reduction of active diaphragmatic contractility using electromyography [25]. Melendez and colleagues assessed respiratory mechanics following thoracotomy using the respiratory inductive plethysmography in patients performing an incentive spirometry. They reported a larger abdominal than ribcage contribution to ventilation before surgery, whilst postoperative findings showed a breathing pattern disorders with increased tidal volume during incentive spirometry resulting from diaphragm derecruitment and greater ribcage recruitment [26]. Maeda et al., observed after thoracotomy a decrease of maximal transdiaphragmatic pressure and ratio of abdominal to transdiaphragmatic pressure, as well as, an increase of intercostal and accessory breathing muscles recruitment and interpreted this as diaphragmatic dysfunction [27]. Only Spadaro et al., used ultrasonography to detected postoperative diaphragm dysfunction. They demonstrated that video-assisted thoracoscopic surgery had a less detrimental impact on diaphragmatic excursion than standard thoracotomy technique. However, the authors assessed only diaphragm movement without estimation of its thickness. In addition, the measurements were performed only during spontaneous breathing and at the latest 24 hours after the surgery [28]. Results of our study added even more knowledge to this issue. First, we noted both procedures were equally linked with decrease of diaphragm inspiratory thickness and magnitude of diaphragm effort (estimated as Diaphragm Thickness Fraction index). Also diaphragm excursion during spontaneous breathing, deep breathing and sniff maneuver was decreased in both groups, but deterioration of movement (measured as Diaphragm Excursion Amplitude Index and Percentage of Diaphragm Excursion Amplitude) was greater after conventional surgery than VATS (Table 1). Furthermore, percentage of patients with postoperative diaphragm paralysis or/and atrophy were significantly smaller after VATS than thoracotomy (Fig 1). These findings emphasize thoracoscopic surgery is less invasive than thoracotomy in terms of diaphragm muscle functioning. It seems greater incision and associated chest wall damage during thoracotomy may play a crucial part in reduction of respiratory efficiency of primary breathing muscle.

In the present paper we also evaluated body static balance parameters after VATS and thoracotomy. We showed both surgical procedures were associated with deterioration of balance parameters, however, patients who underwent conventional thoracotomy had significantly higher postoperative values of two major postural stability indeces: path length and ellipse field (Fig 3). They were also characterized by a greater difference between preoperative and postoperative value in range of this two parameters (Fig 4)—compared to patients who underwent VATS method. We suppose this balance impairment results from postoperative disorders of diaphragm postural function which is inextricably linked with breathing. Properly functioning diaphragm lowers during inhalation providing an optimal intraabdominal pressure (IAP) to stabilization of the lumbar spine and pelvic girdle. Increased respiratory demand, as well as, diminished of diaphragm excursion compromises ability to provide spinal stiffness control [4, 29]. Changes in the respiration pattern also likely to cause greater ambulation of the body’s centre of mass [30]. Disorders of this posturo-respiratory coupling might be confirmed by the results presented in Table 2. We demonstrated a significant relationship: the worse diaphragm excursion, the worse parameters of the path length. In both groups, we observed also a changes in the distribution of feet pressure to the ground in anterior-posterior direction. Before surgery, the greater load on the hindfoot than on the forefoot was demonstrated, while postoperatively more burden was seen on forefoot relative to hindfoot (Fig 5). According to Kolar, abnormal postural activation of diaphragm leads to a greater strain on the ventral region of the spinal column, often resulting in the increased compressive forces on the spine due to compensatory activity of spinal extensors muscles and imbalance between upper and lower chest musculature [31]. We suspect this mechanism lies also at the base of stabilometric changes between forefoot and hindfoot pressure we found. After thoracic surgery, the diaphragm remains higher position what lead to insufficient IAP to proper spine stabilization. As results, compensatory activity of spinal extensors muscles causes an anterior pelvic tilt. Probably this changes in posture causes a displacement of a center of pressure in anterior direction and explain described phenomenon.

According to the data presented in Table 3, each lobe resection was associated with impairment of diaphragm excursion. The greatest postoperative diaphragm movement restriction was identified in patients after upper left and right lobe resection. Independently of resection area, the limitation of mobility was always greater on the left side compared to the right side in each respiratory maneuver. Moreover, one-side resection resulted bilateral diaphragm movement restriction. Sekine and colleagues noted that patients after pulmonary resection of the lower portion of the lung had better lung volume indices than patients after upper lobe resection [32], whilst Takazakura et al. using magnetic resonance imaging reported postoperative diaphragm movement was severely impaired in patients underwent left upper lobectomy or right bilobectomy, moderately impaired in patients underwent right upper lobectomy, right lower lobectomy, or left lower lobectomy, and mildly impaired in those who had underwent right-sided medial lobectomy [33].

In normal condition, left diaphragm dome has a greater excursion than right dome. Difference in range of movement between hemidiaphragms, estimated as side-to-side-variability (STSV) index, should be less than 50%. As shown in Fig 2, in both groups the postoperative values of STSV index were lower than preoperative. The postoperative decrease of this value shows to a bilateral restriction of the diaphragm mobility, regardless of the operated side. These findings are in contrast to the results of Spadaro et al., [28] where the authors stated that diaphragmatic excursion remained unchanged in the nonoperated side. Most likely, the dissimilarity of the findings is a result of a different time of diaphragm excursion measurements after surgery. In our work the postoperative measurements were performed 3–5 days after surgery, while Spadaro et al., conducted measurements 2 hours and 24 hours after surgery. On the basis of our and Spadaro studies, we can speculate that initially postsurgery diaphragm dysfunction is one-sided, but it worsens over time and concern both domes. However, further studies precisely monitored postoperative diaphragm function day by day, are needed to confirm our hypothesis.

Patients after thoracotomy put more pressure on the lower limb on the operated than non-operated side, both with the eyes open and closed. Similarly, patients after the VATS method, but their burden was significantly lower, as well as, the load difference between left and right foot was also smaller after VATS than thoracotomy, independently of resection side (Fig 6). This results stay in accordance with previous study, where Mraz et al., stated that patients after open thoracotomy had a shift of the position of the body’s center and explained it as a protection against pain after chest wall musculature violation [15]. In this point, our results one more time showed less invasivity of VATS lobectomy versus conventional thoracotomy.

Our study has a several limitations. We made a second measurement 3–5 days after surgery, what allows us to draw conclusions about the early postoperative period. Further studies should be performed to a long-term evaluation to assess whether diaphragmatic dysfunction is transient and whether restore of diaphragmatic function is associated with an improvement of equilibrium parameters. In this work, we did not exclude a phrenic nerve injury by measuring the phrenic nerve conduction. However, according to epidemiology data, this type of trauma is very rare after lung cancer resection and should not affect the results. Finally, with caution should be interpreted a results concerning postural sway and diaphragm parameters with regard to area of lobe resection. Due to the small size of the subgroups, the results obtained at this stage should be treated as a preliminary report, and further studies on a larger group of patients are undeniably needed to confirm the findings presented here.

## Conclusions

Thoracic surgery elicits diaphragm dysfunction thereby impairing postural stability. VATS lobectomy was less invasive than lobectomy via thoracotomy in terms of diaphragm muscle function and body balance maintenance parameters.
